# Evaluation of the Use of Sterilized and Non-Sterilized Peruibe Black Mud in Patients with Knee Osteoarthritis

**DOI:** 10.3390/ijerph18041666

**Published:** 2021-02-09

**Authors:** Paulo Fávio Macedo Gouvêa, Zélia Maria Nogueira Britschka, Cristina de Oliveira Massoco Salles Gomes, Nicolle Gilda Teixeira de Queiroz, Pablo Antonio Vásquez Salvador, Paulo Sergio Cardoso Silva

**Affiliations:** 1 Energy and Nuclear Research Institute, Av. Prof. Lineu Prestes 2242, Cidade Universitária, São Paulo 05508-000, SP, Brazil; lamanegradeperuibe@gmail.com (P.F.M.G.); zmnb@uol.com.br (Z.M.N.B.); pavsalva@ipen.br (P.A.V.S.); 2 Department of Pathology, School of Veterinary Medicine and Animal Sciences, University of São Paulo, Av. Prof. Orlando Marques de Paiva, São Paulo 05508-270, SP, Brazil; cmassoco@gmail.com (C.d.O.M.S.G.); nicolle.queiroz@gmail.com (N.G.T.d.Q.)

**Keywords:** Peruíbe Black Mud, knee osteoarthritis, fangotherapy, mud therapy, peloid

## Abstract

This study aimed to evaluate the effects of treatment with Peruíbe Black Mud (PBM) on the clinical parameters and quality of life of patients with knee osteoarthritis and to compare the effects of PBM samples simply matured in seawater and PBM sterilized by gamma radiation. A controlled, double-blind trial was conducted with 41 patients divided into two treatment groups composed of 20 and 21 patients: one group was treated with matured PBM and the other with sterilized PBM. Evaluations were done using the Western Ontario and McMaster Universities Osteoarthritis Index (WOMAC) and the Medical Outcomes Study Short Form 36 (SF-36) questionnaires, the Kellgren and Lawrence (KL) radiographic scale, and the quantification of the serum levels of inflammatory biomarkers. An improvement in pain, physical functions, and quality of life was observed in all of the patients who underwent treatment with both simply matured and sterilized PBM. Nine patients showed remission in the KL radiographic scale, but no statistically significant differences were observed in the serum levels of inflammatory mediators before or after treatment. Peruíbe Black Mud proves to be a useful tool as an adjuvant treatment for knee osteoarthritis (OA), as shown by the results of the WOMAC and SF-36 questionnaires and by the remission of the radiographic grade of some patients on the Kellgren and Lawrence scale.

## 1. Introduction

Fangotherapy, or mud therapy, is the use of mud, slime, or peat, generally known as peloids, for the treatment of musculoskeletal, dermatological, or internal disorders; it is most commonly used for treating articular problems [1,2,3,4]. A peloid is defined as matured mud with healing or cosmetic properties and is composed of a complex mixture of natural fine-grained materials of geological and/or biological origin, mineral or seawater, and usually, organic compounds that show biological metabolic activity [5].

The use of peloids for the treatment of joint disorders has been shown to influence the chondrocyte activity of patients with osteoarthrosis [6,7,8], modulate the production of serum cytokines [9,10,11], and cause a reduction in free radicals and the lipid peroxidation involved in the pathogenesis of chronic and degenerative diseases [12,13]. Apart from these applications, mud therapy has also been used in the treatment of several other diseases [14,15,16,17,18].

Peruíbe Black Mud (PBM) is a peloid found in the city of Peruíbe, which is located in the Southeast of the state of São Paulo, Brazil, and has been used in the treatment of different pathologies, including knee osteoarthritis (OA). Its chemical, mineralogical, and radiological composition was described by Silva et al. [19], and early studies demonstrating its beneficial effects on OA were performed in an experimental rat model [20].

Osteoarthritis (OA) is a chronic, degenerative disease characterized by the progressive loss of joint cartilage. It is one of the main diseases responsible for sick leave from work and early retirement; in fact, when drug and surgical treatments, side effects, loss of joint function, and productivity losses are taken into account, OA is one of the most expensive and limiting diseases [21,22].

Transient changes in cytokine levels involving inflammatory events associated with osteoarthritis have been observed in patients with OA, with effects being especially noted in the concentrations of interleukin 1 (IL-1) and tumor necrosis factor-α (TNF-α) [10]. Studies evaluating the effects of pelotherapy on patients diagnosed with knee OA have shown decreases in serum inflammatory cytokine levels [23,24]. Thus, mud therapy could be an effective complementary treatment for managing OA.

The use of peloids for therapeutic or cosmetic applications cannot be considered exempt from possible adverse health effects a priori, mainly due to the possible presence of pathogenic microorganisms. Therefore, this study investigates the use of peloids sterilized with gamma radiation as another possibility for the treatment of OA. This study aims to verify the efficacy of PBM for treating patients with knee osteoarthritis, as well as to compare the therapeutic effects of PBM prepared with two different processes: simple maturation with seawater and sterilization by gamma radiation after the maturation process. The therapeutic effects were evaluated by applying the Western Ontario and McMaster Universities Osteoarthritis Index (WOMAC) and the Medical Outcomes Study Short Form 36 (SF-36) questionnaires, using the Kellgren and Lawrence (KL) scale based on knee radiographs, and measuring the serum levels of inflammatory mediators: cytokines IL-1, IL-8, IL-6, IL-10 TNF-α, and prostaglandin E_2_ (PGE2).

## 2. Materials and Methods

### 2.1. Patient Characteristics

Before being submitted to treatment, all patients selected to participate in the study received detailed clarifications about the procedures and gave their free and informed consent by signing the consent form for participation in this clinical research. This research was approved by the Brazilian National Commission for Ethics in Research (CONEP) under protocol # 01490012.4.0000.5421.

**Figure 1:**
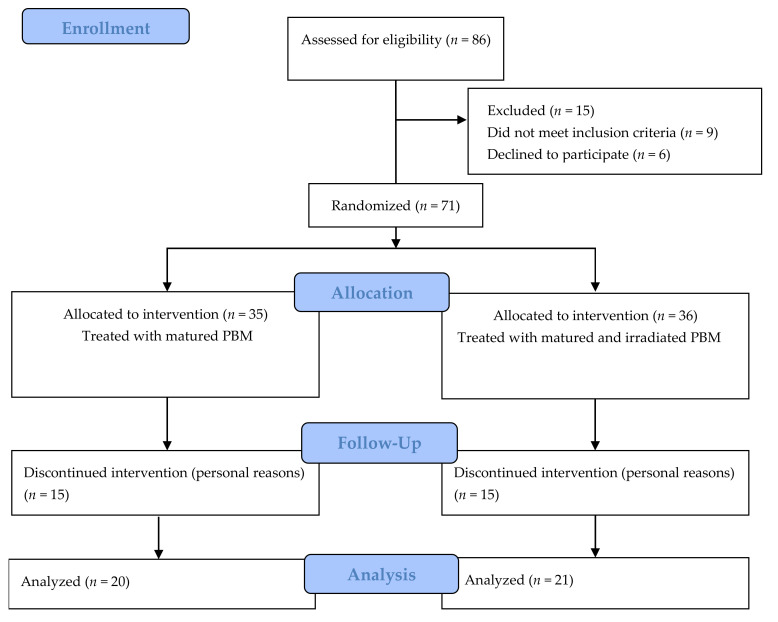
Consort 2010 flow diagram.

A total of 86 patients initially enrolled for participation in the research were interviewed, among which 15 were excluded either due to not meeting all eligibility criteria (not undergoing infiltration during the 6 months prior to the start of the study and not using medications that might affect pathological characteristics—9 patients), or because they declined to participate after the interview (6 patients) (Figure 1). A total of 71 patients were selected to participate in the study: 54 women and 17 men. They were randomly divided into two groups. Of this total, 30 patients discontinued the intervention for different reasons and 41 completed the treatment. The final groups of patients used in the analyses consisted of group A (treated with matured PBM; *n* = 20) and group B (treated with matured and irradiated PBM; *n* = 21).

Patient age varied between 35 and 85 years. The study was conducted using the double-blind method, in which neither the patients nor the treatment providers knew to which group each patient belonged. The clinical and demographic characteristics of the patients at the beginning of treatment are shown in Table 1.

### 2.2. Experimental Design

For randomization, 130 containers containing 25 kg of PBM were sequentially numbered and divided into two groups using a random number generator. One of the two groups of containers was irradiated with gamma rays for sterilization. Patients were distributed into two groups considering their order of inclusion in the study. One group of patients (group A) was treated with PBM collected from the mud deposit and matured for about 1 to 2 months with seawater, which was periodically changed in order to clean and mature the mud. This is referred to simply as “matured mud” in the remainder of this article. The second group (group B) was treated with mud sterilized by gamma radiation after the maturation process, referred to simply as “irradiated mud” below.

For sterilization, the mud was irradiated with a ^60^Co irradiator with a total dose of 25 kGy, using dose rate monitoring. The radiation dose for PBM sterilization was defined according to ISO 11137-2—Sterilization of health care products—radiation [25].

### 2.3. Intervention

The treatment method used was one already in use at the PBM Thermal Complex. It is based on the well-established thermal treatment model that was, according to empirical observations, developed in the city of Peruíbe after years of mud application. It was also based on international experience, mainly from Cuban spas, in which patients undergo a series of treatments consisting of 3 weeks of mud applications followed by 3 weeks of resting, for a total of 15 weeks. The mud was applied on the knee affected by osteoarthritis 5 days a week, for 20 minutes per application, in the form of cataplasm at a temperature of 39 °C [6,14,20].

### 2.4. Evaluation Parameters

#### 2.4.1. Analysis of Pain, Discomfort, and Quality of Life

Patients’ pain, discomfort, and quality of life were evaluated based on subjective information collected by the Western Ontario and McMaster Universities Osteoarthritis Index (WOMAC) and the Medical Outcomes Study Short Form 36 (SF-36) questionnaires.

The WOMAC questionnaire covers the three domains specifically related to patients’ clinical picture: pain (PA), joint stiffness (JS), and the degree of disability for physical functions (PF) [26,27]. The SF-36 questionnaire is much more comprehensive and addresses issues related not only to the patients’ clinical picture but also to their quality of life [28]. The SF-36 has the purpose of examining patients’ own perception of their health and comprises 36 items grouped into 8 dimensions of health: functional capacity (PF), limitations caused by physical problems (RP), limitations due to emotional problems (RE), social functioning (SF), body pain (BP), general health (GH), mental health (MH), and vitality (VT).

The questionnaires were applied at the beginning and the end of the treatment by the same interviewer, at the same facility where the treatment was performed.

#### 2.4.2. Radiographic Evaluation

The radiographic evaluation was done according to the KL [29] classification, which ranks OA severity on a scale ranging from 0 to IV. In this scale, Grade 0 means no signs of OA; Grade I means doubtful signs of the pathology; Grade II means minimal signs of OA; Grade III means moderate signs of the disease; and Grade IV means severe OA conditions. Radiographs were taken at the beginning and the end of each patient’s treatment and evaluated at Clínica São Pedro, in the city of Peruíbe, by a rheumatologist that did not belong to this research group. The radiographs were presented in a random sequence without individual identification of the patients. The radiological score of the patients at the beginning of the treatment is shown in Table 1.

#### 2.4.3. Determination of Serum Concentrations of Cytokines and PGE_2_

To determine serum cytokine and PGE_2_ concentrations, blood samples (5 mL) were taken from the patients’ cubital vein and immediately placed in a Vacutainer tube at the beginning and the end of the treatment. No anticoagulant was added in order to obtain the serum after centrifugation at 500 g and 4 °C. The serum samples were aliquoted in Eppendorf microtubes and stored at −80 °C until the analysis. A human inflammatory cytokines kit (Cytometric Bead Array [CBA]; BD Biosciences, San Jose, CA, USA) was used to quantitatively measure serum concentrations of IL-6, IL-8, TNFα, IL-1β, and IL-10, and a PGE_2_ assay (R&D System, Minneapolis, MN, USA) was used to measure serum concentrations of PGE_2_. All procedures were performed according to the manufacturers’ instructions. Individual cytokine and PGE_2_ concentrations (pg/mL) were computed with specialized software using standard reference curves. Analysis of the serum samples was performed with a FACSCalibur™ cytometer (BD Bioscences) at the Laboratory of Pharmacology and Toxicology, School of Veterinary Medicine and Animal Sciences, University of São Paulo.

### 2.5. Statistical Analysis

The normality of the data was evaluated using the Shapiro–Wilk test. Comparisons between parametric data were performed using t-tests, while comparisons between non-parametric data were performed using the non-parametric Wilcoxon test. A significance level of α = 0.05 was used for all tests; accordingly, “*p*” values < 0.05 were accepted as statistically significant.

## 3. Results

### 3.1. WOMAC Evaluation

Individual scores for the patients’ WOMAC evaluation are shown in Supplementary Material (Table S1). The statistical results for the comparison of patient scores in the WOMAC questionnaire at the beginning and end of the treatment are shown in Table 2 for all patients (T_i_ × T_f_), as well as for groups A (A_i_ × A_f_) and B (B_i_ × B_f_). Results are also presented for comparisons between groups A and B at the beginning (A_i_ × B_i_) and end (A_f_ x B_f_) of the treatment. The comparison between groups shows that the randomization procedure used was effective, as two homogeneous groups were formed regarding the domains addressed by this method of evaluation, i.e., no significant difference between groups was observed before the treatment (*p* > 0.05).

After the treatment, all WOMAC scores were lower than the equivalent scores at the beginning of treatment, with statistically significant differences (*p* < 0.05) found for both groups A and B, and also for the whole group (T), indicating a positive therapeutic response. The results also indicated no differences between the use of matured or irradiated mud in the patients’ perceptions regarding the domains observed by this method (pain, stiffness, and physical function).

### 3.2. SF-36 Evaluation

Individual scores for the SF-36 evaluation are shown in SM1. Table 3 presents the *p*-values for the statistical analysis of the results obtained with the SF-36 questionnaire for the whole group (T) and for groups A and B. It can be observed that the randomization process was effective, forming two homogeneous groups with respect to the domains addressed by this method of evaluation, since the comparison between groups A and B at the beginning of the treatment (A_i_ × B_i_) did not show statistically significant differences (*p* > 0.05), except for the domain “limitations caused by physical problems”. It can also be observed that groups A (A_i_ × A_f_) and B (B_i_ x B_f_) presented a positive therapeutic response for most domains, with the exceptions being “social functioning” in both groups and “mental health” in group B. Nevertheless, a positive response was observed for all domains when considering the whole group (T_i_ × T_f_). In addition, the comparison between groups A and B at the end of the treatment (A_f_ × B_f_) did not produce statistically significant differences.

Similar to what was observed with the WOMAC evaluation, these results indicated no differences between the use of matured or irradiated mud in the patients’ perceptions regarding the observed domains.

### 3.3. Radiographic Evaluation

The results of the KL radiological scores are shown in Table 4 for the whole group of patients (T) and for groups A and B. It can be seen that no patients were classified in Grades 0 (no signs of OA) or I (doubtful) at the start of the treatment. Considering the whole group, nine patients were classified in those grades at the end of the treatment. The number of patients classified in Grade II (minimal) decreased from 27 to 20 between the beginning and end of the treatment, and the number of patients in Grade III (moderate) decreased from 12 to 10. An example of radiographs from the same patient taken at the beginning and end of the treatment, used to reclassify this patient from Grade III to Grade II of the KL scale, is shown in Supplementary Material (Figure S1). The results of the radiographic evaluations indicate that fangotherapy treatment was more effective up to Grade II, while small or no radiographic effects were observed for patients classified in Grades III (moderate) and IV (severe).

### 3.4. Serum Levels of Inflammatory Mediators

The mean values and standard deviations obtained for the concentrations of inflammatory cytokine and PGE_2_ are shown in Table 5. No statistically significant differences were observed between the beginning and the end of the treatment. Moreover, no differences were observed between the groups treated with matured mud and irradiated mud, indicating that irradiating the mud did not change the effectiveness of the treatment.

## 4. Discussion

Evaluating the quality of life of osteoarthritis patients is an important factor for monitoring the evolution of this disease and for obtaining a better interpretation of its impact on the patients’ health. Thus, patient perception is a fundamental variable for reliable OA clinical evaluation and, consequently, for defining therapeutic strategies [30].

In this study, a statistically significant improvement in the patients’ perceptions of their condition between the beginning and the end of the treatment was observed in both groups (treated with matured mud and with irradiated mud) when applying the WOMAC and SF- 36 questionnaires.

The results obtained with the SF-36 questionnaire indicated a statistically significant difference between the treatment groups at the beginning of the treatment in the limitations caused by physical problems (RP). When comparing the characteristics of the patients in each group, it was observed that some members of group B presented more advanced ages, which could explain this difference. However, the results obtained for this domain at the end of the treatment demonstrated a statistically significant improvement in both groups.

No statistically significant differences were found between the beginning and the end of the treatment in the domains of social functioning (SF—for both groups) or mental health (MH—for group B only), which were also evaluated by the SF-36 questionnaire. In the same way that age may have influenced the difference between groups at the beginning of the treatment, regarding the limitations caused by physical problems, this factor may also be the cause of the lack of differences in mental health in group B, which includes older individuals, as this domain places great importance on how individuals imagine their future. Regarding social functioning, no statistically significant differences were observed between the beginning and end of the treatment in any of the groups, suggesting that the health status of individuals significantly affects their interpersonal relationships, and that this is not immediately recovered after the improvement of their clinical status. Furthermore, besides being related to the patients’ general health status, these domains may also be influenced by several other factors.

Although fangotherapy has been used in several parts of the world for centuries [31,32], the mechanism of action of this form of treatment has not yet been fully understood. However, it seems that one of the main effects of this treatment is related to the control of inflammatory processes, since, generally, the most common reasons that lead patients to seek treatment for OA are those associated with inflammatory processes, most often in the chronic phase [14,33,34,35]. Nevertheless, it is possible to suggest some reasons why fangotherapy is advocated and effective for the treatment of knee OA, such as the physical (mechanical and thermal) and chemical characteristics of peloids, as well as the way in which they are applied [34]. In addition to the factors involved in the therapeutic process, the positive effects of fangotherapy may also be related to psychosomatic factors associated with a situation of well-being and a positive state of mind promoted both by the treatment itself and the patients’ withdrawal from their routine, worries, and stress, since at the time of treatment, they are dedicating themselves to treating a disease that generates a great deal of physical and mental discomfort [36].

The joint action of all of these factors produces various effects such as an increase in pain threshold, a reduction in muscle tone [37,38], a reduction in muscle spasms, a increase in serum levels of β-endorphin, and the stimulation of corticotrophin production [39,40].

Recent studies have shown that fangotherapy induces a decrease in circulating levels of prostaglandin E2 (PGE2), leukotrienes B4 (LTB4), interleukin-1β (IL-1β), and tumor necrosis factor-α (TNF-α), which are important mediators of inflammation and pain. In addition, these molecules may act as antioxidants and have immunosuppressive activity [41,42,43].

In this study, nine of the patients with starting radiological indices diagnosed between Grades II and III had their diagnosis after the application of PBM changed to Grades 0 or I, suggesting that mud application may have led to a remission of joint inflammation in the treated knees.

Although several studies have shown the effectiveness of mud pack therapy based on changes in serum concentrations of cytokines and other inflammatory mediators after mud application [6,7,8,9,10,11,12,13], the present study found no changes in the serum levels of PGE2, TNFα, or the interleukins after treatment. The clinical benefits of mud therapy may well be mediated, at least in part, by its systemic anti-inflammatory effects and neuroendocrine-immune regulation in OA. The inflammatory mediators that were quantified represented a circulating status rather than the articular environment since they were measured in the serum and not in the synovial fluid of the patients. This limitation may explain the lack of differences in the concentration of inflammatory mediators before and after treatment in this study. The absence of a significant correlation between the positive results observed with the WOMAC and SF-36 questionnaires, radiographic data, and the serum levels of inflammatory mediators may also have been partly caused by the results being affected by the mean age of patients and/or comorbidities besides OA that were not considered during the experiment.

## 5. Conclusions

The clinical results obtained by patients who underwent the proposed treatments corroborated the use of fangotherapy, in this case, specifically the application of Peruíbe Black Mud, as a useful tool and an adjuvant treatment for knee OA. This was demonstrated by the results of the WOMAC and SF-36 questionnaires and by the remission of the radiographic grade of various patients on the Kellgren and Lawrence scale.

This study demonstrated that the use of PBM was satisfactory in improving both patients’ quality of life and their pain perception.

The study also demonstrated that gamma radiation sterilization did not alter the therapeutic potential of Peruíbe Black Mud.

The limitations of this study, which may be related to the mean age of patients (62 and 63 years for groups A and B, respectively), comorbidities that were not considered during the experiment, and the time of observation after the treatment, may explain the lack of a correlation between the improvements seen with the WOMAC questionnaire, the SF-36 questionnaire, the radiographic scale, and the serum levels of inflammatory mediators. Furthermore, because the main goal of the study was to compare the irradiated with the non-irradiated matured PBM, it was not possible to establish a control group because there was no placebo. Future studies focusing on markers in the synovial fluid are necessary in order to better explore the follow-up treatment options for this disease as well as the follow-up for longer periods.

## Figures and Tables

**Table 1:** Clinical, demographic, and radiological characteristics presented by patients at the beginning of the treatment.

Characteristics	Total	Mean	Median
Number of patients (group A)	20		
Number of patients (group B)	21		
Age—group A (years; mean ± SD)		62 ± 11	62
Age—group B (years; mean ± SD)		63 ± 9	67
Gender—group A (M/F)	5 (25%)/15 (75%)		
Gender—group B (M/F)	6 (29%)/15 (71%)		
Time since first complaint—group A (years; mean ± SD)		6 ± 4	7.5
Time since first complaint—group B (years; mean ± SD)		6 ± 5	2.5
Radiographic scores—group A			
II	14		
III	5		
IV	1		
Radiographic scores—group B			
II	13		
III	7		
IV	1		

**Table 2:** Mean and standard deviation of patient scores in the Western Ontario and McMaster Universities Osteoarthritis Index (WOMAC) evaluation and *p*-values for comparisons between the beginning (i) and end (f) of the treatment for the whole group (T), and intra- and intergroup comparisons (A and B). Results are considered statistically significant for *p* < 0.05.

	Group A (*n* = 20)		Group B (*n* = 21)		*p* Values of Mean Comparisons				
	A_i_	A_f_	B_i_	B_f_					
Domain	Mean ± sd	Mean ± sd	Mean ± sd	Mean ± sd	^1^ T_i_ × T_f_	A_i_ × B_i_	^2^ A_i_ × A_f_	^3^ B_i_ × B_f_	A_f_ × B_f_
PA	9 ± 4	4 ± 4	9 ± 5	5 ± 3	1.3 × 10^−7^	0.82	2.0 × 10^−4^	2.0 × 10^−4^	0.41
JS	3 ± 2	2 ± 2	3 ± 2	2 ± 2	1.5 × 10^−5^	0.78	2.2 × 10^−3^	6.0 × 10^−4^	0.64
PF	28 ± 16	16 ± 16	32 ± 14	19 ± 13	8.0 × 10^−8^	0.58	2.0 × 10^−4^	1.1 × 10^−5^	0.26

^1^ T_i_—whole group at the beginning of the treatment; T_f_—whole group at the end of the treatment; ^2^ A_i_—group A at the beginning of the treatment; A_f_—group A at the end of the treatment; ^3^ B_i_—group B at the beginning of the treatment; Bfgroup B at the end of the treatment. Pain (PA), joint stiffness (JS), and degree of disability for physical functions (PF).

**Table 3:** Mean and standard deviation of patient scores in the Medical Outcomes Study Short Form 36 (SF-36) evaluation and *p*-values for comparisons between the beginning (i) and end (f) of the treatment for the whole group (T), and intra- and intergroup comparisons (A and B). Results are considered statistically significant for *p* < 0.05.

	Group A (*n* = 20)		Group B (*n* = 21)		*p* Values of Mean Comparisons				
	A_i_	A_f_	B_i_	B_f_					
Domains	Mean ± sd	Mean ± sd	Mean ± sd	Mean ± sd	^1^ T_i_ × T_f_	A_i_ × B_i_	^2^ A_i_ × A_f_	^3^ B_i_ × B_f_	A_f_ × B_f_
PF	41 ± 26	62 ± 31	44 ± 30	56 ± 26	9.7 × 10^−7^	0.72	8.3 × 10^−4^	4.3 × 10^−3^	0.56
RP	30 ± 24	53 ± 41	26 ± 33	62 ± 35	2.9 × 10^−5^	0.028	0.010	1.1 × 10^−3^	0.52
BP	35 ± 20	58 ± 25	35 ± 26	53 ± 28	2.5 × 10^−5^	0.96	2.6 × 10^−5^	8.6 × 10^−3^	0.55
GH	58 ± 19	73 ± 24	68 ± 17	78 ± 15	1.5 × 10^−5^	0.10	1.6 × 10^−3^	3.4 × 10^−3^	0.86
VT	52 ± 25	73 ± 21	52 ± 26	72 ± 17	3.3 × 10^−7^	0.87	7.2 × 10^−4^	2.0 × 10^−4^	0.92
SF	69 ± 29	76 ± 30	67 ± 26	77 ± 23	0.034	0.86	0.23	0.070	0.81
RE	37 ± 35	62 ± 42	41 ± 40	62 ± 40	6.7 × 10^−4^	0.75	0.019	0.023	1.0
MH	61 ± 24	71 ± 27	65 ± 22	70 ± 19	6.4 × 10^−3^	0.49	3.0 × 10^−3^	0.25	0.43

^1^ T_i_—whole group at the beginning of the treatment; T_f_—whole group at the end of the treatment; ^2^ A_i_—group A at the beginning of the treatment; A_f_—group A at the end of the treatment; ^3^ B_i_—group B at the beginning of the treatment; B_f_—group B at the end of the treatment. Functional capacity (PF), limitations caused by physical problems (RP), limitations due to emotional problems (RE), social functioning (SF), bodily pain (BP), general health (GH), mental health (MH), vitality (VT).

**Table 4:** Radiological scores according to the Kellgren and Lawrence classification before and after treatment, for the whole group of patients, group A, and group B.

	T_i_	T_f_	A_i_	A_f_	B_i_	B_f_
Grade 0—no signs	0	4	0	3	0	1
Grade I—doubtful	0	5	0	2	0	3
Grade II—minimal	27	20	14	11	13	9
Grade III—moderate	12	10	5	3	7	7
Grade IV—severe	2	2	1	1	1	1

**Table 5:** Mean concentration and standard deviation of measured inflammatory mediators for the groups A and B at the beginning (i) and the end (f) of the treatment.

Mediators	A_i_	A_f_	B_i_	B_f_
IL-1β	1.7 ± 0.3	1.2 ± 0.5	1.2 ± 0.3	0.6 ± 0.3
TNF-α	6.4 ± 1.3	5.3 ± 2.0	6.1 ±1.4	2.4 ± 0.9
IL-6	2.8 ± 0.3	3.0 ± 0.5	3.0 ± 0.3	3.2 ± 0.5
IL-8	12.5 ± 2.5	10.4 ± 2.0	8.4 ± 1.3	10.7 ± 2.0
IL-10	1.8 ± 0.7	1.6 ± 1.0	2.5 ± 1.1	4.1 ± 1.8
PGE2	737 ± 124	633 ± 176	650 ± 158	772 ± 178

## Supplementary Materials

The following are available online at https://www.mdpi.com/1660-4601/18/4/1666/s1, Figure S1: Radiographs of a patient taken at the beginning and end of the treatment, Table S1: WOMAC and SF-36 Scores.
