# Supplementary figures and images for: Evaluation of the Use of Sterilized and Non-Sterilized Peruibe Black Mud in Patients with Knee Osteoarthritis
Source: Int J Environ Res Public Health. 2021 Feb 9;18(4):1666. doi: 10.3390/ijerph18041666 (PMC7916169; doi:10.3390/ijerph18041666)

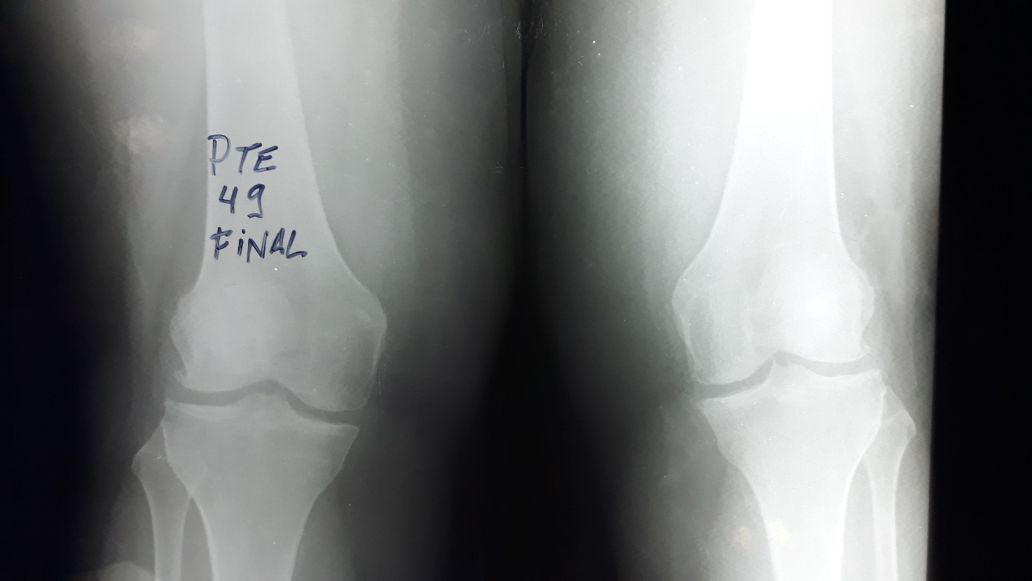

Supplement: Supplementary file 1 [file ijerph-18-01666-s001.zip › Paciente49end.jpg]

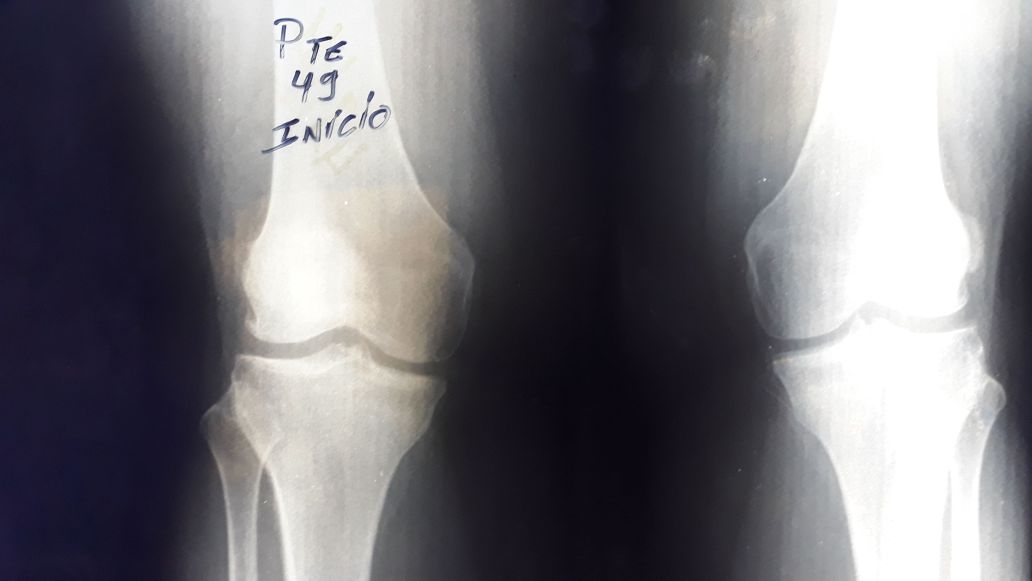

Supplement: Supplementary file 1 [file ijerph-18-01666-s001.zip › Patiente49begining.jpg]
